# Failure or success of search strategies to identify adverse effects of medical devices: a feasibility study using a systematic review

**DOI:** 10.1186/2046-4053-3-113

**Published:** 2014-10-13

**Authors:** Su Golder, Kath Wright, Mark Rodgers

**Affiliations:** 1 Centre for Reviews and Dissemination (CRD), University of York, York YO10 5DD, UK

**Keywords:** Adverse effects, Systematic reviews, Meta-analysis, Information storage and retrieval, Bibliographic databases

## Abstract

**Background:**

Research has indicated that adverse effects terms are increasingly prevalent in the title, abstract or indexing terms of articles that contain adverse drug effects data in MEDLINE and Embase. However, it is unknown whether adverse effects terms are present in the database records of articles that contain adverse effects data of medical devices, and thus, to what extent the development of an adverse effects search filter for medical devices may be feasible.

**Methods:**

A case study systematic review of a medical device was selected. The included studies from a systematic review of the safety of recombinant human bone morphogenetic protein-2 (rhBMP-2) for spinal fusion were used in the analysis. For each included study, the corresponding database record on MEDLINE and Embase was assessed to measure the presence or absence of adverse effects terms in the title, abstract or indexing. The performance of each potential adverse effects search term was also measured and compared.

**Results:**

There were 82 publications (49 studies) included in the systematic review with 51 of these indexed on MEDLINE and 55 on Embase. Ninety-four percent (48/51) of the records on MEDLINE and 95% (52/55) of the records on Embase contained at least one adverse effects related search term. The wide variety of adverse effects terms included in the title, abstract or indexing of bibliographic records, and the lack of any individual high-performing search terms suggests that a combination of terms in different fields is required to identify adverse effects of medical devices. In addition, the most successful search terms differed from the most successful terms for identifying adverse drug effects.

**Conclusions:**

The search filters currently available for adverse drug effects are not necessarily useful for searching adverse effects data of medical devices. The presence of adverse effects terms in the bibliographic records of articles on medical devices, however, indicates that combinations of adverse effects search terms may be useful in search strategies in MEDLINE and Embase. The results, therefore, suggest that not only a search filter for the adverse effects of medical devices is feasible, but also that it should be a research priority.

## Background

Adverse effects are an important consideration in decision-making by patients, clinicians and other health care professionals, policy makers and regulators. An adverse effect is a ‘harmful or undesirable outcome that occurs during or after the use of a drug or intervention for which there is at least reasonable possibility of a causal relation’ [1]. Although adverse effects can occur with any intervention, methodological research into how to find information on adverse effects has, to date, concentrated on identifying adverse effects of drug interventions [2]. The adverse effects of other interventions such as surgery, medical devices, diagnostic tests and physical interventions, however, are equally important. In particular, the importance of the adverse effects of medical devices has been highlighted recently in the press by articles on the potential harm from breast implants and hip prostheses. Although the regulatory process for medical devices is less stringent than for pharmaceutical interventions, the adverse effects of medical devices can be just as serious and can be an important factor in decision-making for health care professionals, policy makers and patients. Identifying the evidence on adverse effects for medical devices is, therefore, paramount to inform health care decisions.

Recent research has indicated that database searching using adverse effects terms will now retrieve the majority of articles on adverse drug effects (92%) [3,4]. However, many of the terms that have proved useful are specific to the adverse effects of drug interventions (for example, adverse drug reaction, chemically induced, toxicity, drug effects, drug induced, drug monitoring and drug hypersensitivity). Similarly, the adverse effects search filters developed to date have also focused on identifying adverse drug effects [2].

A different approach may be required to searching for adverse effects of non-drug interventions, and this is confirmed by a recent study that tested the efficiency of existing search filters in identifying adverse effects papers related to medical devices. While these search filters usually obtain around 92% of the relevant articles in MEDLINE and 89% of the relevant articles in Embase, the same filters only retrieved 74% of the relevant articles related to medical devices in MEDLINE and 54% in Embase [5].

The development of search filters for adverse effects of non-drug interventions and for medical devices, in particular, is therefore a key priority. The first stage of any search filter development should be to assess its feasibility. Search terms related to adverse effects need to be present in the bibliographic database records (in the title, abstract or keywords fields) in order for any article to be potentially retrievable by using adverse effects terms in the database search strategy.

In this case study, we explore the presence or absence of terms related to adverse effects in the bibliographic records of those articles included in a systematic review of a medical device. In addition, we present those terms that are the most useful for identifying the adverse effects of this case study medical device.

## Methods

A case study systematic review of the safety of recombinant human bone morphogenetic protein-2 (rhBMP-2) was selected for analysis [6,7]. Here, rhBMP-2 is a medical device (collagen sponge) that is widely used as an alternative to iliac crest bone graft to promote fusion in spinal surgery. RhBMP-2 is licensed as a medical device with a pharmaceutical component and as with many other medical devices requires a surgical procedure for implementation.

This review was undertaken after a number of sources raised concerns about the safety of rhBMP-2: during the post-marketing period, several non-industry observational studies reported adverse events possibly associated with the use of rhBMP-2; the Food and Drug Administration (FDA) issued a public health notification of potentially life-threatening complications associated with swelling of the neck and throat tissue after rhBMP-2 use in the cervical spine; and a subsequent review of publicly available data suggested an increased risk of complications and adverse events for patients receiving rhBMP-2 that was 10 to 50 times higher than the original randomised controlled trial (RCT) estimates. These sources identified a number of specific adverse events of concern—some dependent upon spinal location—such as dysphagia, retrograde ejaculation, heterotopic bone formation and osteolysis. These possible adverse effects of rhBMP-2 have received much publicity, and a high-quality systematic review encompassing all the evidence was required to answer the many ambiguities. The Centre for Reviews and Dissemination (CRD) at the University of York was commissioned by Yale University to conduct such as a review. The systematic review of the safety of rhBMP-2 included a search of the following databases: BIOSIS Previews (1969–2008 only), CENTRAL, Embase, MEDLINE, PubMed, Science Citation Index (SCI), ClinicalTrials.gov, the Database of Abstracts of Reviews of Effects (DARE), the Health Technology Assessment (HTA) database and ToxFile. PubMed was selected in addition to MEDLINE as PubMed includes citations not included in MEDLINE as well as the MEDLINE database itself. In addition to database searching, various supplementary search methods were used that included reference checking; contacting authors of key papers; publishing a call for evidence in Spine Journal, The Back Letter newsletter and on the Internet and setting up automated ‘current awareness’ searches in Zetoc Alert from the British Library and in MEDLINE to notify us whenever new data matching our search criteria were loaded onto the databases. The broad search strategy contained just two facets: rhBMP-2 and spinal fusion with multiple synonyms, textwords and indexing terms used for each facet. No adverse effects terms were applied to the search strategy in order to try to maximise sensitivity and allow a prospective analysis of the potential performance of adverse effects terms if they had been included. A pragmatic decision was made to include disease terms (spinal fusion) although it was appreciated that this may have missed papers which referred to adverse effects of the intervention in other bone conditions, or where spinal fusion was not mentioned. No study design or date limits were applied, and the full search strategy is published elsewhere [6,7].

### Inclusion criteria

All studies (RCTs and observational studies) of more than ten adult participants that compared rhBMP-2 with any other spinal fusion technique and reported adverse effects were eligible for inclusion in the systematic review.

### Analysis

The included references from this case study systematic review formed the basis of the analysis. This review contained 82 publications representing 49 studies. Of the 82 publications, 40 referred to 13 RCTs and one single-armed study and the other 42 publications referred to 35 observational studies.

The first stage of the analysis was to check whether each paper was listed in MEDLINE or Embase. MEDLINE and Embase were selected as they are the most commonly searched databases in systematic reviews and have been evaluated for the availability of adverse drug effects terms in the previous research [3,4,8]. In order to ascertain whether each paper was contained in the databases, several iterations using author names and words from the title were used.

### Adverse effects terms in the database records

For each database, the available papers were sorted according to the following criteria:

1. The authors mentioned terms synonymous with ‘adverse effects’ in the title or abstract, potentially enabling the papers to be found in an electronic search. Generic adverse effects terms, such as ‘adverse events’, ‘side effects’, ‘complications’, ‘safe’ and ‘risk’ were accepted.

2. The authors mentioned specific named adverse effects terms such as ‘swelling’, ‘dysphagia’ or ‘blood loss’ in the title or abstract.

3. The papers had been indexed (using subject headings or subheadings) with relevant terms for adverse effects, potentially enabling the papers to be found in an electronic search. Generic adverse effects terms were accepted on the basis that they could be considered synonymous with ‘adverse effects’. Examples of included indexing terms are ‘postoperative complications/’ in MEDLINE and ‘device safety/’ in Embase. Examples of included subheadings are ‘complications (co)’ in MEDLINE and ‘side effect (si)’ in Embase.

4. The papers had been indexed with specific named adverse effects terms such as ‘airway obstruction/’ in MEDLINE or Embase, ‘dysphagia/’ in Embase, and ‘bleeding/’ in Embase.

## Results

### Included records

There were 51 of the 82 included references on MEDLINE (five were not identified at the time of the original searches) and 55 of the 82 included references on Embase (four were not identified at the time of the original searches). Seven of these included references from Embase were duplicated within in Embase, and for the purposes of this analysis, the duplicate records were not included.

### Adverse effects terms by search fields

The percentage of relevant records with either generic or specific named adverse effects terms in the bibliographic records (title, abstract or indexing) in MEDLINE and Embase is presented in Table 1. The most commonly occurring adverse effects terms in the MEDLINE records were generic adverse effects terms in the abstract (73%), followed by specific adverse effects terms in the abstract (61%) and then generic adverse effects subheadings (51%). In Embase, the most commonly occurring adverse effects terms were specific named adverse effects in the indexing (73%), followed by generic adverse effects terms in the abstract (71%) and then specific adverse effects terms in the abstract (67%).

**Table 1:** Adverse effects terms in the bibliographic records in MEDLINE or Embase

	**Relevant MEDLINE records retrieved (*N *=51)**	**Relevant Embase records retrieved (*N *=55)**
Generic adverse effects terms		
Title	16% (8)	16% (9 plus 1 duplicate)
Abstract	73% (37)	71% (39 plus 4 duplicates)
Title or abstract	73% (37)	71% (39 plus 4 duplicates)
Indexing	31% (16)	35% (19)
Subheadings	51% (26)	62% (34)
EMBASE section headings	NA	16% (9)
Any generic adverse effects terms	88% (45)	85% (47 plus 5 duplicates)
Specific adverse effects terms		
Title	18% (9)	20% (11 plus 1 duplicate)
Abstract	61% (31)	67% (37 plus 4 duplicates)
Title or abstract	61% (31)	67% (37 plus 4 duplicates)
Indexing	37% (19)	73% (40 plus 3 duplicates)
Any specific adverse effects terms	69% (35)	82% (45 plus 5 duplicates)
Any adverse effects terms in any field	94% (48)	95% (52 plus 5 duplicates)

Overall, a search with generic adverse effects search terms would retrieve 88% of the available records in MEDLINE and 85% of the available records in Embase. Searching with specific named adverse effects terms would perform better in Embase than in MEDLINE retrieving 92% of the records in Embase compared with 69% in MEDLINE.

A search with both generic and specific named adverse effects in either MEDLINE or Embase would retrieve a high proportion of the records available in each of the databases (94% and 95%, respectively).

### Individual search terms

The highest number of relevant records in MEDLINE was retrieved by the subheading ‘adverse effects (ae)’ (47%), followed by ‘complication’ in the abstract (31%), the indexing term ‘postoperative complications/’ (27%), ‘complications’ in the abstract (27%), ‘safety’ in the abstract (27%), ‘safely’ in the abstract (27%) and then ‘blood loss’ in the abstract (20%). A complete listing of the performance of individual search terms in MEDLINE is presented in Additional file 1: Table S1.

The highest number of relevant records in Embase was retrieved by the subheading ‘complication (co)’ (49%), followed by the term ‘complications’ in the abstract (31%); the indexing term ‘pseudarthrosis/’ (24%); the subheading ‘adverse drug reaction (ae)’ (22%); the terms ‘blood loss’, ‘complications’ and ‘safety’ in the abstract (18%) and then the indexing terms ‘bleeding/’ and ‘dysphagia/’ (18%). A complete listing of the performance of individual search terms in Embase is presented in Additional file 2: Table S2.

## Discussion

This case study demonstrates the feasibility of developing a generic search filter for adverse effects of medical devices. Eighty-eight percent of MEDLINE records and 85% of Embase records contained at least one generic searchable term for adverse effects. With the addition of specific named adverse effects terms, search performance could potentially reach 94% in MEDLINE and 95% in Embase.

The most successful individual adverse effects search terms varied from those identified in similar research using adverse drug effects case examples. There was a greater tendency for terms such as ‘complication(s)’, ‘safe’, ‘safely’ or ‘safety’ to identify articles on the adverse effects of medical devices than articles on drug effects. The wide disparity of potentially useful search terms and the lack of any one term successfully retrieving the majority of articles would suggest that any filter will need to be developed using a large set of relevant records to ensure that the optimal combination of search terms is captured.

The main difference between MEDLINE and Embase was the higher number of specific adverse effects terms presents in Embase. While this undoubtedly improves the sensitivity of Embase searches, it may be to the detriment of the precision of Embase searches. The trade-off between sensitivity and precision in database searching is inevitable, and any search filter development will need to consider this trade-off.

The relatively high proportion of records with specific named adverse effects in the abstract or in the indexing in Embase is reassuring given that adverse effects are often a secondary or even tertiary outcome in primary studies. Previous research in 2001 indicated that adverse drug effects terms are not present in the title, abstract or indexing nearly a quarter of all relevant research and this problem is more likely to be an issue in articles of non-drug interventions [8]. This finding led to recommendations that searchers should not rely on adverse effects terms [9]. However, since 2001, there have been improvements in the reporting of adverse effects in the title, abstract or indexing in primary studies related to drug interventions [4]. This may have resulted from efforts such as the CONSORT extension for harms [10], calls from the Cochrane Adverse Effects Methods Group (CAEMG) and in the published literature [11-33]. These improvements may extend to the reporting in articles on medical devices.

Interestingly, the results of this case study indicate that adverse effects terms are more likely to be present in the bibliographic records for medical devices than for drug interventions [4,8]. However, it should be recognised that this case study systematic review included many recent articles and improvements in adverse effects reporting have been identified over time [4]. It should also be noted that these results might also be affected by the level of caution demonstrated during the screening process in this case study systematic review and the same level of attention may not be present during routine screening of systematic review search results.

### Limitations

This analysis is only based on one case study systematic review. This limits the generalisability of the results. This was an unusual review in that the authors were able to obtain unpublished data directly from the manufacturer. The included studies also included an unusually high number of conference abstracts and multiple publications for the same study (particularly for the clinical trials). The medical device is also rather atypical for a device in that it has a pharmaceutical component. Thus, our findings may be less generalizable.

Analysis of the precision of the search terms would have been of interest, however, was beyond the scope of this case study.

The usefulness of searching with specific terms is measured here, but this will only be applicable to those reviews in which the searchers have a clear idea beforehand what the potential key events are. This approach is not as useful for unexpected or new events. However, in this case study review, we have also demonstrated the value of generic adverse effects terms which can be used in any review. More research is required using more case study systematic reviews. Nevertheless, our evaluation is an important first step to guide systematic reviewers in an area which is likely to gain greater prominence, given the heightened public interest in safety of devices following recent scares.

## Conclusions

This case study demonstrates the presence of adverse effects terms in the bibliographic records of articles on medical devices. The search terms most successful in identifying adverse effects of medical devices differed from those used in adverse effects search filters for drug interventions. Consequently, creating a search filter for adverse effects of medical devices is feasible and should be a research priority.

## Competing interests

The authors declare that they have no competing interests.

## Authors’ contributions

SG conceived the idea, wrote the protocol, carried out the analysis and wrote the first and subsequent drafts of the paper. KW conducted the original searches in the systematic review and commented and contributed to all stages of the paper. MR carried out all stages in the original review, from sifting the records, data extraction and analysis. MR also helped ascertain the included studies for this paper and commented and contributed to all versions of the paper. All authors read and approved the final manuscript.

## Supplementary Material

Additional file 1: Table S1 Records retrieved by individual search terms in MEDLINE. Click here for file

Additional file 2: Table S2 Records retrieved by individual search terms in Embase. Click here for file
