# Supplementary material for: Failure or success of search strategies to identify adverse effects of medical devices: a feasibility study using a systematic review
Source: Syst Rev. 2014 Oct 13;3:113. doi: 10.1186/2046-4053-3-113 (PMC4203467; doi:10.1186/2046-4053-3-113)
Supplement: Additional file 1: Table S1 — Records retrieved by individual search terms in MEDLINE. [file 2046-4053-3-113-S1.docx]

## Additional table 1: Records retrieved by individual search terms in MEDLINE

| **Generic Adverse Effects Terms in the Title** | **Number of MEDLINE records** |
| --- | --- |
| Complications | 6 |
| Safe | 1 |
| Safety | 1 |
| **Generic Adverse Effects Terms in the Abstract** | **Number of MEDLINE records** |
| Complication | 16 |
| Complications | 11 |
| Safety | 11 |
| Safely | 11 |
| Safe | 6 |
| Risk | 6 |
| Adverse events | 6 |
| Adverse effects | 3 |
| Adverse reactions | 2 |
| Adverse event | 1 |
| Untoward effects | 1 |
|  |  |
| **Generic Adverse Effects Indexing Terms** |  |
| Postoperative complications | 14 |
| Risk factors | 3 |
| Safety | 1 |
|  |  |
| **Subheadings** |  |
| Adverse effects | 24 |
| Complications | 4 |
| Chemically induced | 4 |
|  |  |
| **Specific Adverse Effects Terms in the Title** | **Number of MEDLINE records** |
| Airway obstruction | 3 |
| Heterotopic bone formation | 1 |
| Radiculitis | 1 |
| Pancreatic cancer | 1 |
| Renal insufficiency | 1 |
| Retrograde ejaculation | 1 |
| Subsidence (same paper as osteolysis) | 1 |
|  |  |
| **Specific Adverse Effects Terms in the Abstract** | **Number of MEDLINE records** |
| Blood loss | 10 |
| Dysphagia | 7 |
| Pseudarthrosis | 5 |
| Hoarseness | 4 |
| Airway obstruction | 3 |
| Dyspnea | 3 |
| Radiculitis | 3 |
| Subsidence | 3 |
| Swelling | 3 |
| Wound infection | 3 |
| Hematomas | 2 |
| Heterotopic bone formation | 2 |
| Osteolysis | 2 |
| Wound complications | 2 |
| Bone regrowth | 1 |
| Discomfort | 1 |
| Donor site pain | 1 |
| Edema | 1 |
| Fracture | 1 |
| Hematoma formation | 1 |
| Inflammatory reactions | 1 |
| Neurologic deficit | 1 |
| Ossification | 1 |
| Renal insufficiency | 1 |
| Retrograde ejaculation | 1 |
| Wound complication | 1 |
|  |  |
| **Specific Adverse Effects Indexing Terms** | **Number of MEDLINE records** |
| Osteogenesis | 6 |
| Pain, Postoperative | 4 |
| Airway Obstruction | 3 |
| Deglutition Disorders | 2 |
| Hoarseness | 2 |
| Pseudarthrosis | 2 |
| Aphasia | 1 |
| Dyspnea | 1 |
| Ejaculation | 1 |
| Ossification, Heterotopic | 1 |
| Pancreatic Neoplasms | 1 |
| Radiculopathy | 1 |
