# Supplementary material for: Failure or success of search strategies to identify adverse effects of medical devices: a feasibility study using a systematic review
Source: Syst Rev. 2014 Oct 13;3:113. doi: 10.1186/2046-4053-3-113 (PMC4203467; doi:10.1186/2046-4053-3-113)
Supplement: Additional file 2: Table S2 — Records retrieved by individual search terms in Embase. [file 2046-4053-3-113-S2.docx]

## Additional table 2: Records retrieved by individual search terms in Embase

| **Generic Adverse Effects Terms in the Title** | **Number of Embase records** |
| --- | --- |
| Complications | 6 (plus one duplicate) |
| Adverse event | 1 |
| Adverse events | 1 |
| Risk | 1 |
| Safe | 1 |
| **Generic Adverse Effects Terms in the Abstract** | **Number of Embase records** |
| Complications | 17 (plus one duplicate) |
| Complication | 10 (plus one duplicate) |
| Safety | 10 |
| Adverse adj2 events (adverse swelling events) | 8 (plus one duplicate) |
| Risk | 8 |
| Safe | 7 (plus two duplicates) |
| Adverse events | 7 (plus one duplicate) |
| Adverse effects | 2 |
| Adverse reactions | 2 |
| Safely | 2 |
| Adverse event | 1 |
| Risks | 1 |
| Untoward effects | 1 |
| **Generic Adverse Effects Indexing Terms** |  |
| Postoperative complication | 11 |
| Drug safety | 3 |
| Risk | 2 |
| Absence of side effects | 1 |
| Chemically induced disorder | 1 |
| Device safety | 1 |
| Drug induced disease | 1 |
| Patient safety | 1 |
| Safety | 1 |
| Unspecified side effect |  |
| **Subheadings** |  |
| Complication | 27 |
| Adverse Drug Reaction | 12 |
| Side effect | 9 |
| Drug toxicity | 1 |
| **Embase Section Headings** |  |
| Adverse Reactions Titles | 9 |
|  |  |
| **Specific Adverse Effects Terms in the Title** | **Number of Embase records** |
| Airway obstruction | 2 |
| Radiculitis | 2 |
| Heterotopic bone formation | 1 (plus one duplicate) |
| Dysphagia | 1 |
| Osteolysis | 1 |
| Pancreatic cancer | 1 |
| Renal insufficiency | 1 |
| Retrograde ejaculation | 1 |
| Subsidence (same paper as osteolysis) | 1 |
| Swelling | 1 |
|  |  |
| **Specific Adverse Effects Terms in the Abstract** | **Number of Embase records** |
| Blood loss | 10 (plus 3 duplicate) |
| Dysphagia | 8 (plus one duplicate) |
| Pseudarthrosis | 7 (plus one duplicate) |
| Radiculitis | 5 |
| Subsidence | 5 |
| Swelling | 5 |
| Osteolysis | 4 |
| Hoarseness | 3 (plus one duplicate) |
| Donor site pain | 2 (plus one duplicate) |
| Heterotopic bone formation | 2 (plus one duplicate) |
| Ossification | 3 |
| Airway obstruction | 2 |
| cyst formation | 2 |
| Discomfort | 1 (plus one duplicate) |
| Donor-site morbidity | 2 |
| Dyspnea | 2 |
| Edema | 2 |
| Retrograde ejaculation | 2 |
| Wound complications | 2 |
| Wound infection | 2 |
| Bone regrowth | 1 |
| Donor-site infection | 1 |
| Excessive bone formation | 1 |
| Fracture | 1 |
| Hematoma formation | 1 |
| Hematomas | 1 |
| Inflammatory reactions | 1 |
| Neurologic deficit | 1 |
| Renal insufficiency | 1 |
| Wound complication | 1 |
|  |  |
| **Specific Adverse Effects Indexing Terms** | **Number of Embase records** |
| Pseudarthrosis | 13 |
| Bleeding | 10 |
| Dysphagia | 10 |
| Infection | 6 |
| Intervertebral disk hernia | 6 |
| Hoarseness | 4 |
| Radiculitis | 4 |
| Spine disease | 4 |
| Degenerative disease | 3 |
| hematoma | 3 |
| Heterotopic ossification | 3 |
| Bone swelling | 2 |
| Drug induced cancer | 2 |
| dyspnea | 2 |
| Injury | 2 |
| Neoplasm | 2 |
| Neurologic disease | 2 |
| Radiculopathy | 2 |
| Retrograde ejaculation | 2 |
| Swelling | 2 |
| wound complication | 2 |
| Wound infection | 2 |
| Acute airway obstruction | 1 |
| Airway obstruction | 1 |
| Arthritis | 1 |
| Bone erosion | 1 |
| Bone infection | 1 |
| Bursitis | 1 |
| Drug fatality | 1 |
| Edema | 1 |
| Fever | 1 |
| Fracture | 1 |
| Fracture nonunion | 1 |
| Graft dysfunction | 1 |
| Joint swelling | 1 |
| Mortality | 1 |
| Nerve injury | 1 |
| Neurological complication | 1 |
| Neuropathic pain | 1 |
| Osteoarthritis | 1 |
| Pancreas cancer | 1 |
| paresthesia | 1 |
| Postoperative hemorrhage | 1 |
| Postoperative infection | 1 |
| Sepsis | 1 |
| Seroma | 1 |
| Surgical infection | 1 |
| Tarlov cyst | 1 |
| Vertebra fracture | 1 |
| Weakness | 1 |
